# Deep learning and radiomics-based system for early diagnosis of hip synovitis in juvenile idiopathic arthritis

**DOI:** 10.3389/fimmu.2025.1689862

**Published:** 2026-01-16

**Authors:** Jun Kou, Chunmei Yin, Yang Gao, Daozhen Huang, Chunjiang Yang, Huan Xiao

**Affiliations:** 1 Department of Ultrasound, Children’s Hospital of Chongqing Medical University, National Clinical Research Center for Child Health and Disorders, Ministry of Education Key Laboratory of Child Development and Disorders, Intelligent Application of Big Data in Pediatrics Engineering Research Center of Chongqing Education Commission of China, Chongqing, China; 2 Department of Ultrasound, Yibin Hospital Affiliated to Children’s Hospital of Chongqing Medical University, Yibin, Sichuan, China

**Keywords:** deep learning, hip synovitis, juvenile idiopathic arthritis, musculoskeletal ultrasound, radiomics

## Abstract

**Objective:**

Juvenile Idiopathic Arthritis (JIA) frequently affects children’s hips, causing severe progression, but early hip synovitis lacks obvious symptoms and is hard to detect via conventional ultrasound, delaying diagnosis. magnetic resonance imaging (MRI), though accurate, is costly and inaccessible for routine use. This study aims to develop an automatic identification system for the early diagnosis of hip synovitis in JIA through the integration of deep learning and radiomics techniques.

**Methods:**

A YOLO-JIA model specifically designed for the automatic segmentation of hip ultrasound images was developed. Radiomic features were extracted from these segmented regions. Subsequently, feature selection was performed using the analysis of variance (ANOVA) test followed by least absolute shrinkage and selection operator (LASSO) regression. Based on the selected features, a Random Forest (RF) classification model was constructed and evaluated separately on an internal and an external validation set.

**Results:**

The YOLO-JIA model demonstrated high precision (0.98) and recall (1.00) in object detection tasks, with a mean average precision at 50–95% (mAP50–95) for mask (M) reaching 0.86. The RF classification model achieved an area under the curve (AUC) of 0.88 on the internal validation set and 0.81 on the external validation set. Decision curve analysis further confirmed the clinical utility of our proposed system. Finally, the models were integrated and deployed locally.

**Conclusion:**

This study successfully developed a system for the early diagnosis of JIA hip synovitis based on deep learning and radiomics. The system offers an effective and reliable means for early screening, enhancing diagnosis rates, and ultimately reducing the risk of severe joint damage in JIA patients.

## 1 Introduction

Juvenile Idiopathic Arthritis (JIA) was one of the most prevalent chronic rheumatic diseases in childhood and a leading cause of acquired disabilities in children. Global epidemiological studies revealed that its prevalence ranged from 3.8 to 400 per 100,000 individuals (1). The hip joint, serving as the central hub for weight-bearing and movement in the human body, was affected in up to 20% - 60% of JIA patients (2, 3), and hip involvement often indicated a more severe disease burden and a poorer prognosis (4). Persistent joint synovitis not only led to cartilage destruction and bone erosion but also interfered with normal skeletal development, resulting in premature epiphyseal closure and femoral neck deformities (5, 6). Once these structural changes occurred, they were difficult to fully reverse even after inflammation was controlled, ultimately potentially necessitating total hip arthroplasty in patients (7). Therefore, early identification and intervention for hip synovitis were crucial for preventing irreversible structural damage and improving long-term outcomes. However, the early diagnosis of hip synovitis in JIA was confronted with multiple challenges due to the absence of obvious clinical symptoms in affected children, limited ability of children to express their symptoms, and the lack of specificity in laboratory indicators (8). Although magnetic resonance imaging (MRI) was recognized as the gold standard for evaluating hip synovitis (9), it was expensive, time-consuming, and had limited accessibility. Ultrasound, being convenient, economical, and radiation-free, could directly display anatomical structural abnormalities at the site of synovial attachment. Nevertheless, early inflammatory changes were often subtle and difficult to visually distinguish on ultrasound (10).

In recent years, breakthroughs had been made in deep learning within the field of medical image analysis, presenting new opportunities for ultrasound medicine in disease diagnosis and prognosis evaluation (11). By learning features from raw data and conducting automatic analysis, deep learning demonstrated unique advantages when processing ultrasound image data (12). Meanwhile, radiomics was capable of extracting a large number of quantitative features from medical images, capturing subtle changes that were difficult to discern with the naked eye (13). The combination of deep learning and radiomics had shown promising application prospects in the diagnosis of various diseases (14, 15). However, artificial intelligence solutions specifically targeting hip ultrasound in JIA remained a gap.

In view of this, this study integrated deep learning and radiomics techniques with the aim of developing an automatic identification system for hip synovitis in JIA. This system precisely located the lesion area by constructing an automatic segmentation module for hip ultrasound images. Subsequently, it extracted and screened key radiomic features to quantify lesion information. Finally, a diagnostic model for hip synovitis was established to achieve effective disease identification. This study aimed to provide clinicians with a timely, cost-effective, and non-invasive tool for hip joint assessment, effectively compensating for the limitations of traditional ultrasound evaluation. It would offer objective auxiliary decision-making support for clinicians and contribute to optimizing the allocation of medical resources.

## 2 Materials and methods

### 2.1 Patient selection

This prospective diagnostic study recruited pediatric patients who presented to the Children’s Hospital of Chongqing Medical University between January 2024 and December 2025. All enrolled patients underwent concurrent MRI and hip ultrasound examinations at presentation. The study cohort was categorized strictly according to the MRI reference standard for the presence or absence of hip synovitis. The abnormal group comprised patients with MRI-confirmed hip synovitis, all of whom fulfilled the International League of Associations for Rheumatology (ILAR) classification criteria [16] for Juvenile Idiopathic Arthritis (JIA). The control group consisted of patients with no imaging evidence of synovitis on MRI, which included healthy children and pediatric patients with other non-inflammatory musculoskeletal conditions.

Inclusion criteria were as follows: (1) age ≤ 16 years; (2) presented with hip pain, limited range of motion, or other clinical suspicion warranting an MRI examination for synovitis assessment; (3) complete clinical and imaging data; (4) the time interval between MRI and ultrasound examinations did not exceed 3 days. Exclusion criteria were: (1) presence of other autoimmune diseases; (2) anticipated poor compliance with the study; (3) failure to provide informed consent; (4) Evidence of irreversible structural joint damage, such as bone erosions, cartilage loss, or joint space narrowing.

To ensure the robustness and generalizability of the findings, an independent external validation cohort was prospectively collected from the Yibin Hospital of the Children’s Hospital of Chongqing Medical University, applying the same rigorous inclusion and MRI-based classification criteria. The study was approved by the Ethics Committee of Children’s Hospital of Chongqing Medical University (approval number: 2023-490), and informed consent forms were signed by all subjects and their legal guardians.

### 2.3 Image acquisition

Ultrasound examinations were conducted using GE Logiq 11 and Logiq e ultrasound systems (GE Healthcare), which were equipped with high-frequency linear array transducers (7–18 MHz, models L4-12t, L8-18i, ML6-15, and L2-9). The pediatric patients were placed in a standard position: lying supine with their feet externally rotated. The operator positioned the transducer at the anterior inferior iliac spine to obtain cross-sectional images of the acetabular labrum-joint capsule region above the femoral head. The image acquisition parameters were optimized as follows: the resolution ranged from 1024×768 to 2048×1536 pixels, the dynamic range was set between 60–80 dB, and the scanning depth was adjusted individually according to the patient’s body size.

MRI examinations were performed using a GE Discovery MR 750 3.0T system. The scanning sequences included coronal and axial T1FSE, T2 fat-suppressed FSE, and contrast-enhanced scans. The MRI diagnostic criteria were based on the recommendations of the European Society of Musculoskeletal Radiology regarding the use of magnetic resonance imaging in rheumatic diseases (17). All images were independently and double-blindly evaluated by two radiologists with more than 10 years of experience.

### 2.4 YOLO - JIA model

Based on the collected hip ultrasound images, two experienced senior musculoskeletal ultrasound physicians used LabelMe software to conduct pixel - level annotation of the joint capsule area. Subsequently, the annotated dataset was randomly divided into a training set and a validation set in an 8:2 ratio. To standardize data specifications and enhance the model’s generalization ability, all images were standardized and uniformly resized to 512×512 pixels. Meanwhile, a systematic data augmentation strategy was implemented, which specifically included random rotation (within an angle range of ±15°), random scaling (with a scaling factor of 0.8 - 1.2 times), random horizontal/vertical flipping, brightness adjustment (within an amplitude of ±20%), and contrast variation (within an amplitude of ±20%).

In terms of model construction, a YOLO - JIA model specifically designed for the analysis of JIA hip ultrasound images was developed based on the YOLO11 architecture. During the model training process, an early - stopping mechanism (patience = 100) was incorporated to optimize the training process and prevent overfitting. The final model was primarily evaluated based on mean average precision at 50–95 (mAP50–95) for mask(M), and its performance was comprehensively assessed using the validation set.

### 2.5 Feature extraction, selection, and modeling

Using the finally preserved YOLO-JIA model, automatic region of interest (ROI) segmentation was performed on the grouped ultrasound images; following manual review to ensure segmentation accuracy, quantitative features were extracted from the verified ROIs using the PyRadiomics library. These features were of diverse types, covering first order features, shape features, gray level co-occurrence matrix (GLCM) features, gray level size zone matrix (GLSZM) features, gray level run length matrix (GLRLM) features, neighboring gray tone difference matrix (NGTDM) features, and gray level dependence matrix (GLDM) features. For detailed information on the extracted features, refer to our previously published literature (18).

Subsequently, the dataset was randomly divided into a training set and a validation set in an 8:2 ratio. In the feature selection stage, a two-stage selection strategy was adopted. First, the analysis of variance (ANOVA) test was employed to screen out features with significant inter-group differences (p < 0.05). Then, least absolute shrinkage and selection operator (LASSO) regression combined with 10-fold cross-validation was used for further dimensionality reduction, and the “one standard error rule” (1 - SE rule) was applied to determine the optimal regularization parameter λ, thereby selecting the final feature subset.

Based on preselected clinical features, a Random Forest (RF) classification model was first developed using the internal training cohort, and its initial performance was evaluated on the internal validation set. Subsequently, an independent external validation cohort was prospectively assembled to rigorously assess the model’s generalizability and robustness across clinically heterogeneous populations. Concurrently, decision curve analysis (DCA) was conducted to quantify the net clinical benefit across a range of clinically meaningful risk thresholds, thereby validating the model’s practical utility. Ultimately, the optimized final model was deployed as a prototype system to facilitate real - time clinical decision - making support.

### 2.6 Statistical analysis

Python 3.10 and PyTorch 2.5.0 (with CUDA 12.5 support) were employed for deep learning model development, leveraging the computational capabilities of an NVIDIA GeForce RTX 4090 GPU. ANOVA was utilized to evaluate intergroup differences across three cohorts, with statistical significance defined as p < 0.05. Radiomic feature extraction was performed using the Pyradiomics package. Feature selection via ANOVA and LASSO regression, along with RF classification, were implemented using standard functionalities from the Pandas, NumPy, Scikit-learn, and Matplotlib libraries.

## 3 Result

### 3.1 Overview of research

**Figure 1:**
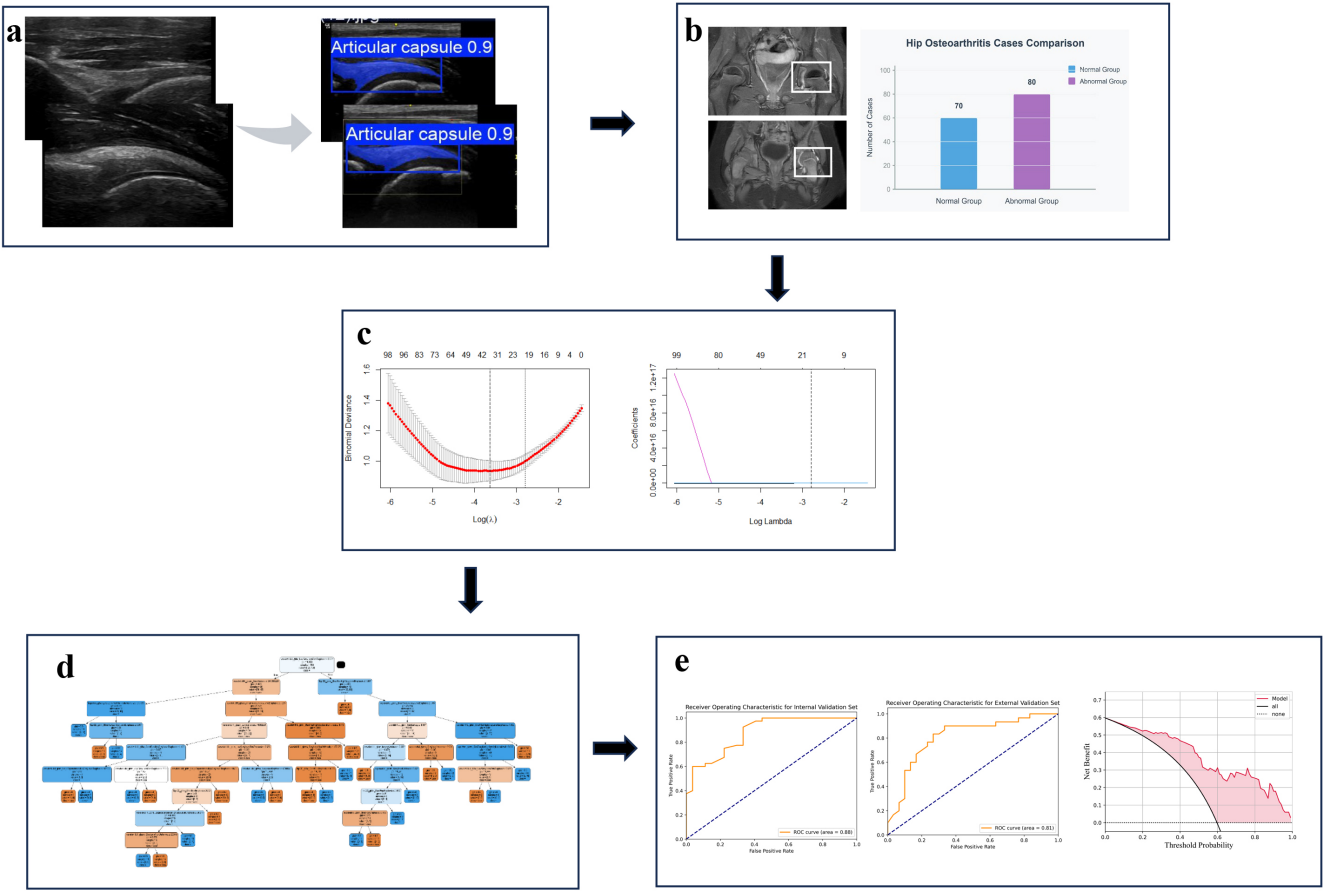
Flowchart of the Automatic Identification and Diagnosis Model Establishment for JIA hip synovitis ultrasound images. **(a)** Automatic segmentation of ultrasound images via the YOLO-JIA model; **(b)** Classification based on MRI gold-standard results; **(c)** Feature Selection through LASSO Regression; **(d)** Modeling with Random Forests; **(e)** Assessment of RF Model Performance;.

This study utilized a total of 524 high-quality hip ultrasound images, derived from two prospectively enrolled cohorts: a primary cohort of 150 pediatric patients (80 MRI-positive, 70 MRI-negative) for model development, and an independent external validation cohort of 60 patients (30 per group) to test generalizability. The YOLO-JIA model was successfully constructed and employed for automatic segmentation. A total of 1,422 radiomic features were extracted from the segmented joint capsule regions. Following preliminary screening via ANOVA, LASSO regression was applied for dimensionality reduction, ultimately selecting 22 key features. The Random Forest classification model, built based on these features, demonstrated favorable diagnostic performance on the validation set. In the end, the optimized model was deployed as a prototype for real-time clinical support. The complete research workflow is illustrated in **Figure 1**.

### 3.2 YOLO-JIA evaluation

**Figure 2:**
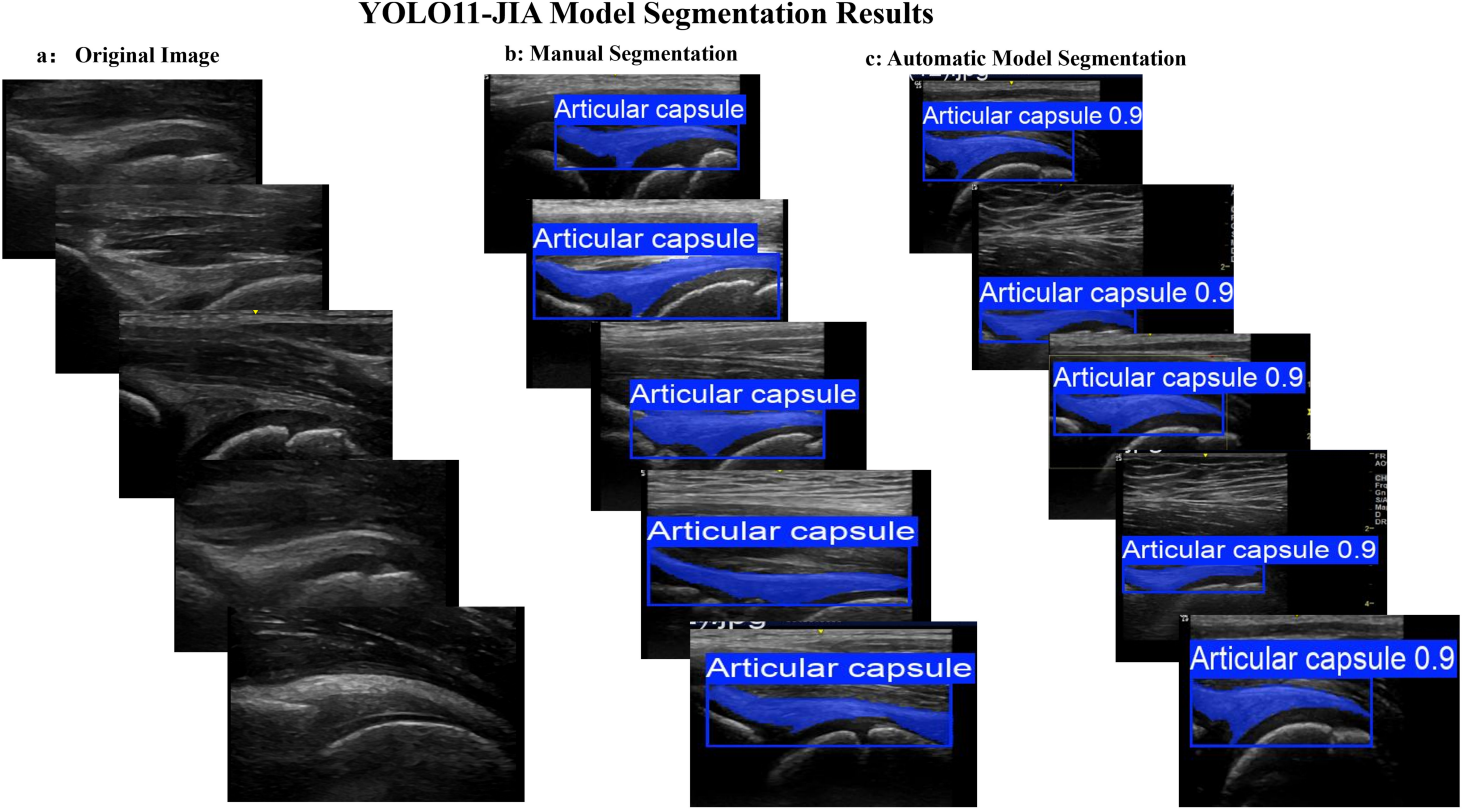
Example of hip joint ultrasound image segmentation results. **(a)** Original ultrasound image; **(b)** Manual segmentation result; **(c)** YOLO-JIA automatic segmentation result.

As shown in **Table 1**, the best-performing model on the validation set demonstrated exceptional performance in object detection, achieving a precision of 0.98 and a recall of 1.0. For semantic segmentation tasks, Precision(M)) reached a perfect score of 1.0, while Recall(M) was as high as 0.99. Notably, under the more stringent mAP50-95(M) evaluation metric, the model maintained a high score of 0.86, fully validating its robustness. Additionally, the model required only approximately 10.46 ms to process a single image, underscoring its significant clinical applicability. **Figure 2** displays the segmentation results across different cases, revealing a high degree of consistency between the automatic segmentation outputs of YOLO-JIA and the manual segmentations performed by experts. For every image in the synovitis prediction set, the generated ROIs were accurately localized and their contours conformed to the expert-defined standards.

**Table 1:** Evaluation metrics of the optimal model.

Category	Metrics	Value
Detection	Precision (B)	0.98
	Recall (B)	1.00
	mAP50 (B)	0.99
	mAP50-95 (B)	0.95
Segmentation	Precision (M)	1.00
	Recall (M)	1.00
	mAP50 (M)	1.00
	mAP50-95 (M)	0.86
Speed	Preprocess/ms	1.04
	Inference/ms	6.67
	Postprocess/ms	2.75

M, Mask; B, Boundary.

### 3.3 Feature selection and model evaluation

**Figure 3:**
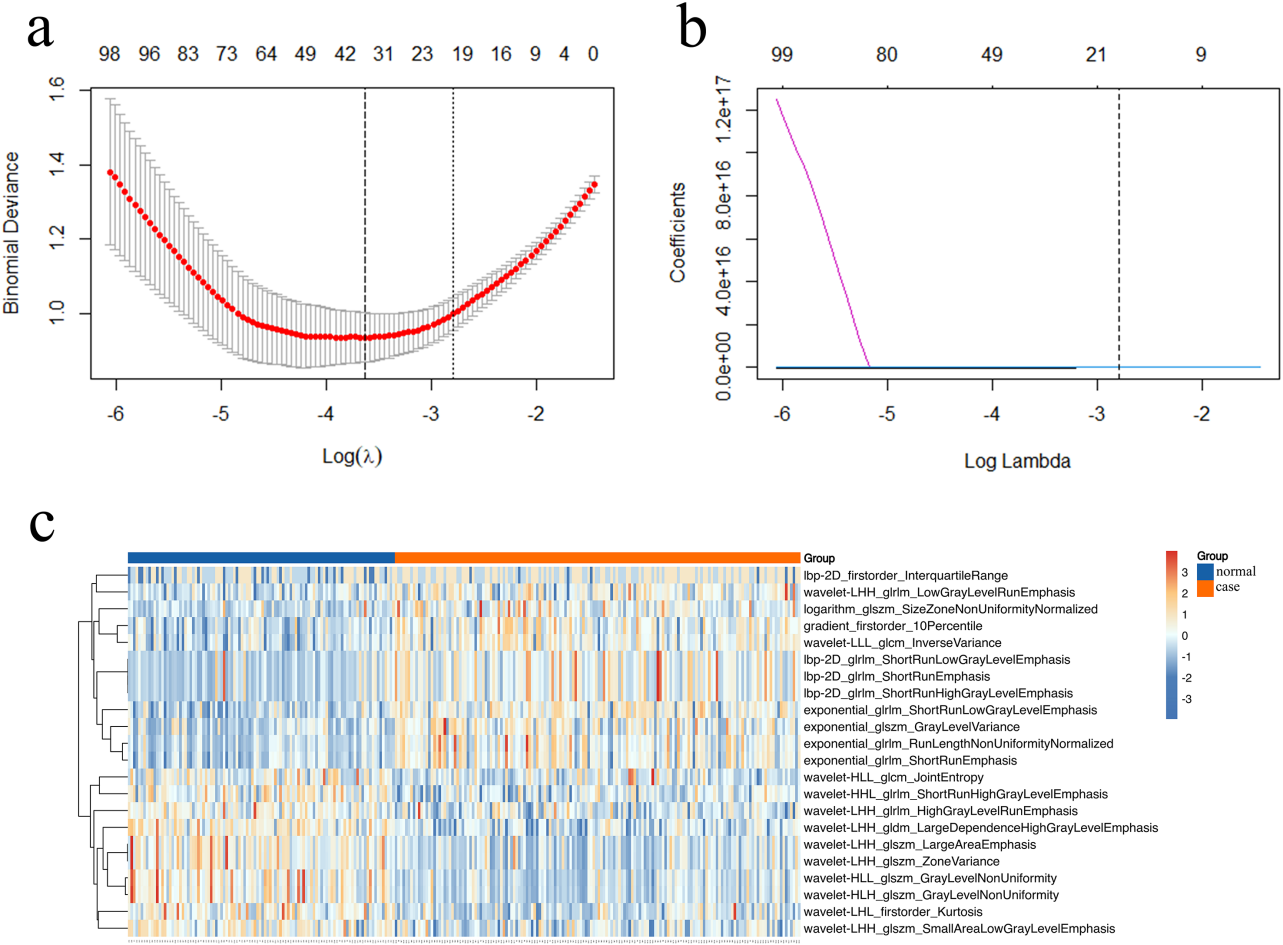
LASSO regression and feature cluster heatmap. **(a)**: the MSE-Lambda Relationship: Lambda at One Standard Error, **(b)**: Feature Selection Variation with Lambda, **(c)**: the Cluster Heatmap of the Selected 22 Features.

During the feature selection phase, the relationship curve between the Lambda regularization parameter and Binomial Deviance was plotted (**Figure 3a**), revealing a typical U-shaped distribution. Based on this distributional characteristic, the optimal Lambda value was selected using the “1-SE rule,” thereby narrowing down the initial pool of 1,422 raw features to 22 key features with significant discriminative power (**Figure 3b**). To further validate the discriminative capacity of the selected features, cluster heatmap analysis was conducted (**Figure 3c**). In the heatmap, red regions indicated high feature expression within specific groups, while blue regions denoted moderate-to-low expression. The results demonstrated pronounced clustering effects, strongly confirming the high discriminative efficacy of the selected features in diagnosing JIA.

**Figure 4:**
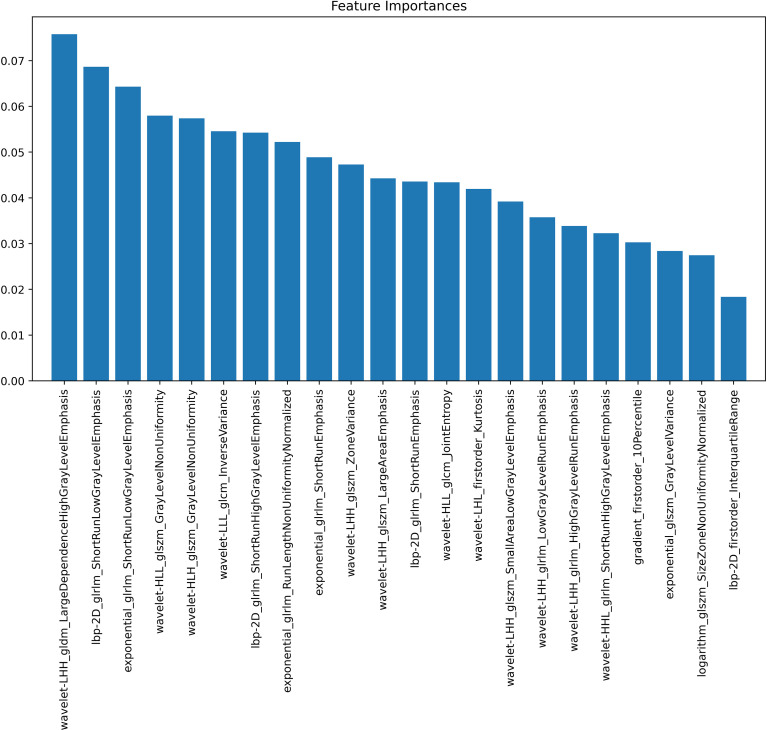
Feature importance scores from Random Forest analysis.

**Figure 5:**
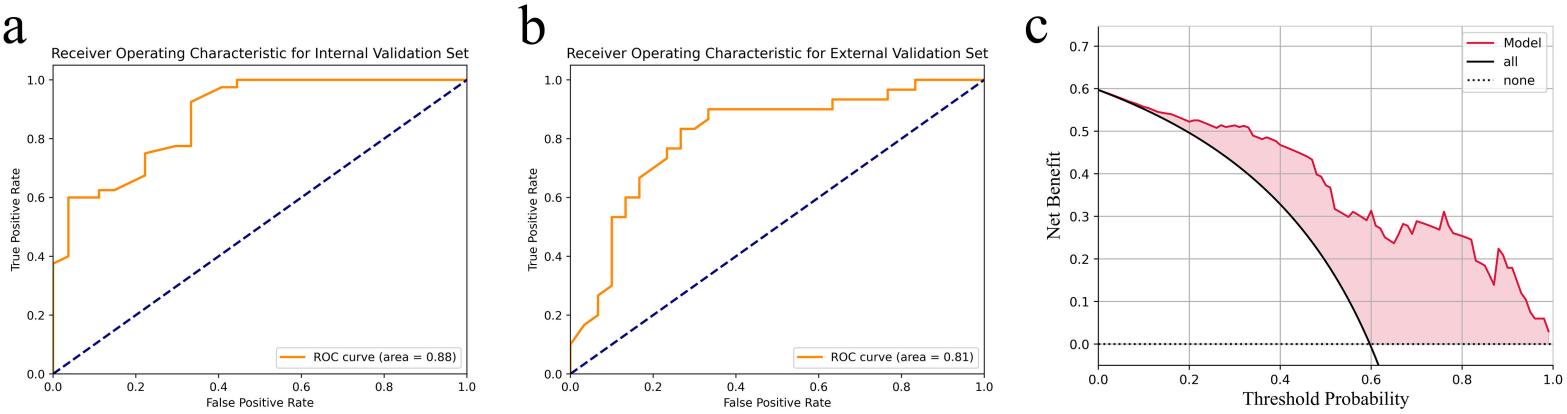
Model validation and clinical utility assessmentm. **(a)** ROC curve of the Internal Validation Set; **(b)** ROC curve of the External Validation Set; **(c)** Decision Curve Analysis.

**Figure 6:**
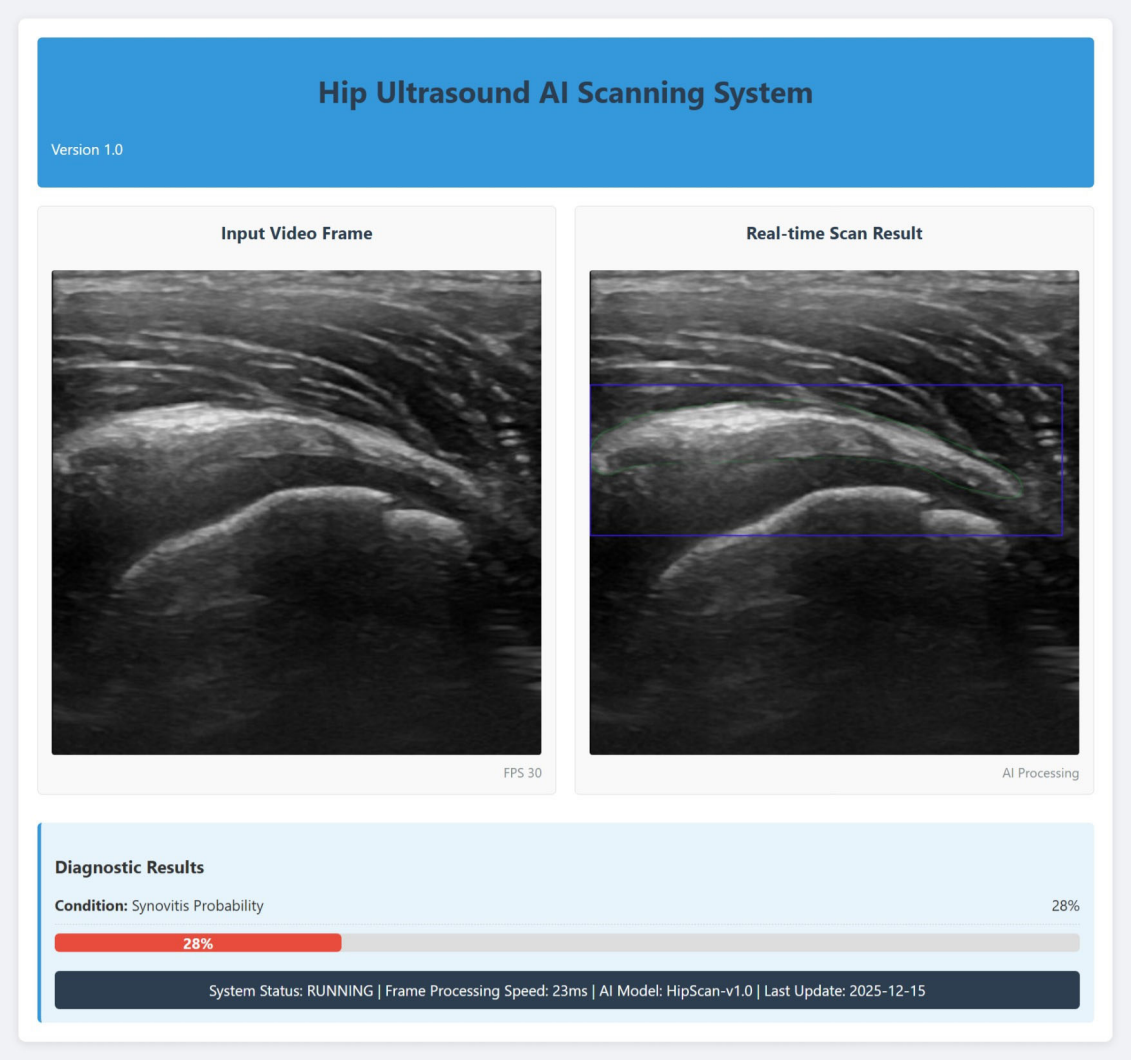
Hip ultrasound AI scanning system: Real-time frame-by-frame analysis and synovitis risk assessment.

Feature importance analysis, based on the weight values depicted in **Figure 4**, was performed to understand the contribution of individual radiomic features. This analysis facilitated the selection of a concise, high-impact feature subset for constructing the final, more efficient random forest classifier. The detailed component tree diagrams of the Random Forest model, which complement the feature importance analysis, are provided in **Supplementary File 1** for clarity and completeness. As shown in **Figure 5**, the model demonstrated excellent diagnostic performance on the internal validation set, with an area under the ROC curve (AUC) of 0.88 (**Figure 5a**), indicating high overall discriminative accuracy. On the independent external validation set, the model achieved an AUC of 0.81 (**Figure 5b**), confirming its maintained discriminative ability and generalizability on unseen data, as well as its potential for clinical translation. Furthermore, decision curve analysis (DCA) results (**Figure 5c**) showed that across a wide range of threshold probabilities, the net clinical benefit of using the proposed model for decision−making consistently exceeded that of the “treat all” and “treat none” strategies, underscoring its practical clinical utility. As shown in **Figure 6**, a localized deployment of the constructed artificial intelligence model was successfully achieved, and a prototype Hip Ultrasound AI Scanning System (Version 1.0) was developed. This system is capable of processing ultrasound video streams in real time, with AI-processed results dynamically displayed and a synovitis risk assessment concurrently output. The real-time performance and engineering feasibility of the proposed model were validated in an actual clinical scanning environment through this prototype, laying the groundwork for subsequent hardware−software integration aimed at clinical application.

## 4 Discussion

This study integrated deep learning and radiomics techniques to construct the YOLO-JIA intelligent detection model for hip joint ultrasound. The model demonstrated favorable performance across various metrics on the validation set, providing an efficient and reliable auxiliary tool for the early diagnosis of hip synovitis in JIA. This technology combines the benefits of ultrasonography (non - invasive, fast, and economical) with the efficient analysis ability of artificial intelligence. It captured and quantified subtle imaging changes that are difficult to discern with the naked eye, while overcoming issues such as high operator dependency and difficulties in selecting standard imaging planes inherent in traditional ultrasound assessments. Additionally, the system featured fast processing, simple operation, and low cost, making it suitable for primary healthcare institutions. It contributed to improving the allocation of medical resources, advancing the construction of a tiered diagnosis and treatment system for hip synovitis in JIA, and providing a reliable decision-support tool for clinical practice.

YOLO11, with its lightweight design, avoided the issues of high computational overhead and overfitting risk caused by complex structures (19), significantly enhancing processing efficiency (20). This architectural characteristic, which balances speed while ensuring accuracy, made YOLO highly compatible with medical ultrasound image analysis tasks (21). In this study, the YOLO11 architecture was introduced into the field of hip joint ultrasound image analysis, and the results demonstrated that high-precision automatic recognition of joint capsules was achieved. During the same phase, a UNet model was also trained for joint capsule recognition (**Supplementary File 2**). However, its performance proved inadequate for this specific task, with an IoU of only 77.84%, Dice/F1-score of 87.54%, and a precision of 0.67, reflecting poor segmentation accuracy, low boundary alignment, and notable false-positive rates. Additionally, its post-processing time reached 123.00 ms, creating a substantial efficiency bottleneck. In contrast, the proposed YOLO-based model achieved near-perfect metrics across all indicators. This disparity arises primarily from architectural suitability: YOLO’s end-to-end detection-segmentation framework, enhanced with multi-scale fusion and attention mechanisms, enables precise localization and segmentation of blurred synovial boundaries in ultrasound images (22, 23), yielding high precision (mAP50-95: 0.86) and efficiency (post-processing: 2.75 ms). In comparison, the symmetric encoder-decoder design of UNet loses fine-grained details during down-sampling, lacks explicit localization capability, and depends on computationally heavy post-processing—limitations that collectively render it unsuitable for real-time, high-precision applications such as hip ultrasound synovial analysis (24).

In terms of feature screening, a two-stage feature selection strategy combining ANOVA and LASSO regression was employed. ANOVA was capable of precisely identifying feature subsets with significant differences between two groups, effectively reducing feature noise in subsequent modeling processes (25, 26). LASSO regression, through its L1 regularization mechanism, automatically compressed the coefficients of redundant or irrelevant features to zero, thereby selecting the most representative variables from highly correlated feature sets (27). Ten-fold cross-validation was adopted to determine the optimal regularization parameter λ, and the “1-SE rule” was integrated to mitigate the risk of overfitting caused by direct modeling with small-sample, high-dimensional data (28). This approach also significantly enhanced the efficiency and stability of biomedical feature selection (29), facilitating a deeper understanding of the imaging manifestation mechanisms of hip synovitis (30). The combined application of ANOVA and LASSO achieved complementary advantages, avoiding the issue of focusing solely on individual feature significance while neglecting interactions among features (31, 32). Compared to single-method approaches, the multi-stage feature screening strategy was more robust (33) and significantly improved the stability of ultrasonic radiomic features (34).

To improve the interpretability of the radiomics model, a pathophysiological analysis was performed on the 22 selected key features, which can be grouped into four distinct categories, each corresponding to specific imaging and pathological characteristics of synovial inflammation. The first category comprises short-run texture features, such as exponential_glrlm_ShortRunEmphasis, lbp-2D_glrlm_ShortRunHighGrayLevelEmphasis, and wavelet-LHH_glrlm_LowGrayLevelRunEmphasis. These reflect focal, speckle-like echo patterns, which may correspond to microcalcifications or dense inflammatory infiltrates (high-intensity short runs) or to regions of microvascular proliferation and edema (low-intensity short runs), collectively indicating microscale heterogeneity in synovial tissue. The second category consists of regional heterogeneity features, including exponential_glszm_GrayLevelVariance, logarithm_glszm_SizeZoneNonUniformityNormalized, and wavelet-HLL_glszm_GrayLevelNonUniformity. These quantify the spatial variability in echo intensity and zone size within the synovium. Elevated values suggest a patchy distribution of active inflammatory, proliferative, and fibrotic areas, serving as imaging markers of inflammatory activity. The third category involves regional scale and structural features, exemplified by wavelet-LHH_glszm_LargeAreaEmphasis and wavelet-LHH_gldm_LargeDependenceHighGrayLevelEmphasis. These provide information on the extent of synovial involvement: the former may indicate widespread synovial thickening or effusion, while the latter could reflect confluent fibrotic changes associated with chronic disease. The fourth category covers multi-scale texture features such as wavelet-LHL_firstorder_Kurtosis and wavelet-HLL_glcm_JointEntropy. Derived from wavelet transforms, these capture textural variations across specific frequency and orientation bands. For example, increased wavelet-HLL_glcm_JointEntropy values may indicate heightened textural complexity due to tissue boundary disruption from edema or hyperplasia. Taken together, these features provide a multi-dimensional quantitative representation of synovial inflammation—encompassing local texture irregularity, spatial heterogeneity, structural extent, and multi-scale complexity—thereby grounding the model’s decisions in recognizable pathophysiological concepts and enhancing its clinical interpretability and trustworthiness.

The RF algorithm not only effectively captured the synergistic changes among multiple features and understood the complex nonlinear relationships between feature variables and the target variable (35), but also exhibited low sensitivity to variations in feature scales (36). Additionally, based on Gini coefficient-based importance evaluation, this algorithm comprehensively reflected the integrated contributions of features across multiple decision paths (37), providing necessary interpretability support for clinical applications. It is noteworthy that in the test set, the model displayed relatively high sensitivity but comparatively low specificity, potentially giving rise to false-positive results when applied independently. This trade-off, however, is justifiable in the context of clinical screening and early diagnosis, where early signs of synovitis are often subtle and atypical. High sensitivity ensures a low miss rate, which is crucial for conditions such as Juvenile Idiopathic Arthritis (JIA), where early intervention is vital. False positives can be addressed through subsequent evaluations and follow-ups, while missed diagnoses could significantly delay treatment. Therefore, positioning the model as an “auxiliary screening tool” rather than a definitive diagnostic tool makes its current performance an acceptable compromise. In future work, to improve specificity, the labeled dataset is planned to be expanded and refined through the inclusion of more easily confused negative samples.

More importantly, to highlight the necessity of the proposed framework, several alternative models were also constructed and systematically evaluated. As shown in **Table 1 of Supplementary File 3**, the YOLO-based direct binary classification model performed poorly in both detection and segmentation tasks, with its mAP50–95 being lower than 0.69 and recall failing to exceed 0.84. This reflected issues with inadequate localization accuracy and a high false-negative rate. **Table 2 of Supplementary File 3** further demonstrated that the UNet-based segmentation and classification model performed even worse, with an IoU below 42.95% and a Dice coefficient no higher than 60.09%. These results indicated fundamental flaws in boundary localization and pixel-level segmentation, and the post-processing time exceeded 124.70ms, failing to meet real-time requirements. As shown in **Table 1 of Supplementary File 4**, the ResNet152-based direct image classifier, although achieving a high recall rate for abnormal classes, exhibited catastrophic failure on the normal class (with a recall rate of only 0.10), highlighting severe class imbalance and clinical unreliability. The common limitation of these single-stage or direct classification methods lies in their architecture, which struggles to achieve real-time processing while maintaining high precision and balanced class sensitivity—particularly when confronted with challenges such as blurred tissue boundaries, low contrast, and significant anatomical variability in ultrasound images. In contrast, the two-stage framework proposed in this study decouples precise localization/segmentation from diagnostic classification, allowing each module to be optimized for its specific sub-task. This design not only overcomes the shortcomings of single models in segmentation accuracy, class balance, and inference speed, but also establishes a systematic, robust, and clinically feasible diagnostic process that meets real-time demands. In addition, the two-stage framework developed in this study has been locally implemented as an integrated hip ultrasound AI scanning system, establishing a closed-loop workflow from image acquisition to real-time analysis. The system enables frame-by-frame visualization of segmentation results, allowing operators to evaluate automated delineations against anatomical knowledge in real time. When necessary, scanning techniques can be adjusted immediately for on-the-fly correction. This approach significantly reduces analytical bias arising from the accumulation of segmentation errors, thereby enhancing the robustness of the method and its reliability in clinical practice.

It should be acknowledged that this study has several limitations. The sample size is relatively modest, and most of the data was primarily derived from a single center. Although preliminary validation at an independent external center demonstrated the model’s potential for generalization, JIA is a highly heterogeneous disease. The current sample may not fully capture this diversity, and the lack of subgroup analyses limits insights into performance across different patient profiles. Consequently, the generalizability of the model to broader, real-world populations requires further verification. To address these limitations, future translational strategies should adopt a multidimensional approach, including conducting multi-center clinical validation in prospective cohorts to assess the real-world generalizability. Collaborations with ultrasound device manufacturers to integrate workflows and develop usability engineering solutions, such as software-hardware integration or independent workstation development with user-friendly interfaces, will be essential.

## 4 Conclusion

This study successfully developed a deep learning- and radiomics-based early diagnosis system for JIA hip synovitis, achieving an end-to-end technical pipeline from automated ultrasound image segmentation to synovitis diagnosis. The system provided a feasible clinical tool for early screening, enhancing early diagnosis rates, reducing risks of severe joint damage, and offering reliable screening for primary healthcare facilities. These advancements facilitated rational allocation of medical resources, improved overall JIA management, and served as a valuable reference for clinical translation of Artificial Intelligence in pediatric imaging.

## Data Availability

The raw data supporting the conclusions of this article will be made available by the authors, without undue reservation.
