# Supplementary figures and images for: Deep learning and radiomics-based system for early diagnosis of hip synovitis in juvenile idiopathic arthritis
Source: Front Immunol. 2026 Jan 16;16:1689862. doi: 10.3389/fimmu.2025.1689862 (PMC12855055; doi:10.3389/fimmu.2025.1689862)

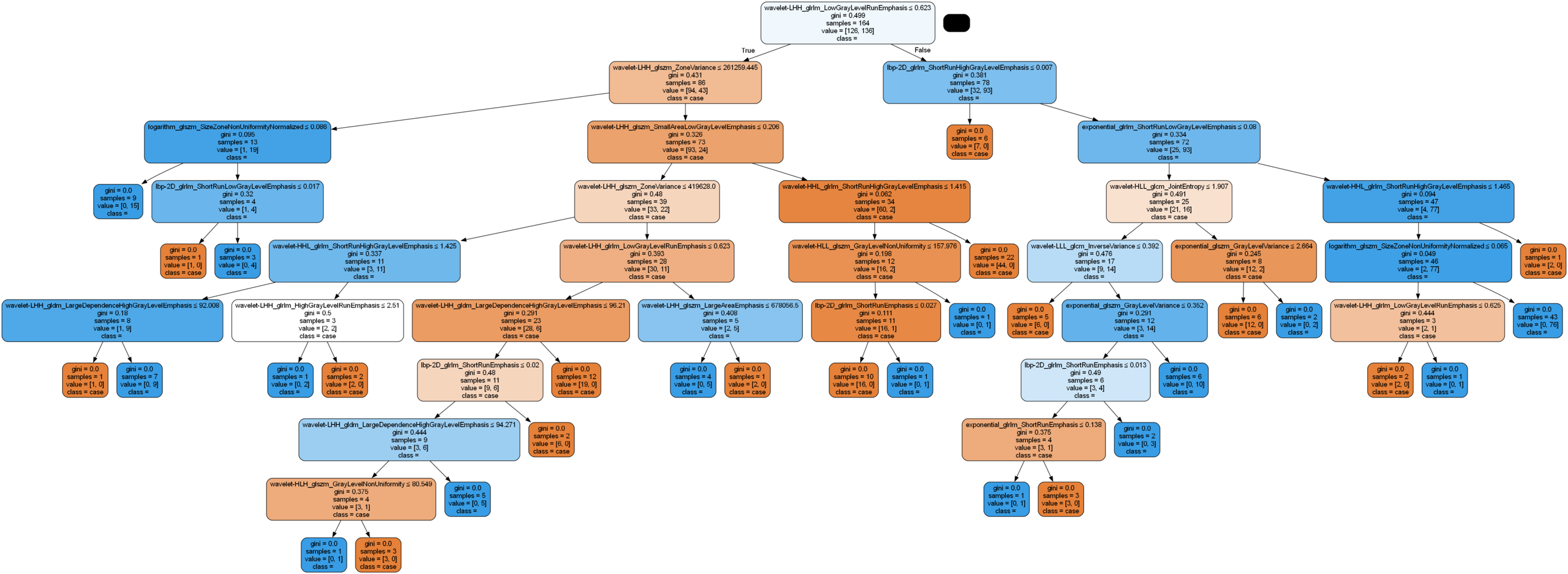

Supplement: Supplementary file 1 [file DataSheet1.pdf]
