# Supplementary material for: Deep learning and radiomics-based system for early diagnosis of hip synovitis in juvenile idiopathic arthritis
Source: Front Immunol. 2026 Jan 16;16:1689862. doi: 10.3389/fimmu.2025.1689862 (PMC12855055; doi:10.3389/fimmu.2025.1689862)
Supplement: Supplementary file 2 [file DataSheet2.pdf]

Table 1 Performance Evaluation of UNet Model Joint Capsule Segmentation in Ultrasound Images

Metrics	Value
IoU	77.84
Acc	89.01
Dice	87.54
F1-score	87.54
Precision	0.67
Recall	0.77
Preprocess /ms	5.80
Postprocess/ms	123.00
