# Supplementary material for: Deep learning and radiomics-based system for early diagnosis of hip synovitis in juvenile idiopathic arthritis
Source: Front Immunol. 2026 Jan 16;16:1689862. doi: 10.3389/fimmu.2025.1689862 (PMC12855055; doi:10.3389/fimmu.2025.1689862)
Supplement: Supplementary file 3 [file DataSheet3.pdf]

Table1 Performance Evaluation of YOLO Model in Joint Capsule Segmentation with Concurrent Synovitis Detection

	Metrics	Normal Group	Case Group
Detection	Precision (B)	0.87	0.88
	Recall (B)	0.82	0.84
	mAP50 (B)	0.93	0.91
	mAP50-95 (B)	0.64	0.68
Segmentation	Precision (M)	0.87	0.88
	Recall (M)	0.82	0.84
	mAP50 (M)	0.92	0.91
	mAP50-95 (M)	0.60	0.63
Speed	Preprocess /ms	1.10	
	Inference /ms	5.50	
	Postprocess/ms	3.20	

Table2 Performance Evaluation of the UNet Model in Joint Capsule Segmentation with Concurrent Synovitis Detection

Metrics	Normal Group	Case Group
IoU	34.66	42.95
Acc	52.71	55.88
Dice	51.47	60.09
F1-score	51.47	60.09
Precision	50.30	64.98
Recall	52.71	55.88
Preprocess/ms	5.30	
Postprocess/ms	124.70	
