# Supplementary material for: Deep learning and radiomics-based system for early diagnosis of hip synovitis in juvenile idiopathic arthritis
Source: Front Immunol. 2026 Jan 16;16:1689862. doi: 10.3389/fimmu.2025.1689862 (PMC12855055; doi:10.3389/fimmu.2025.1689862)
Supplement: Supplementary file 4 [file DataSheet4.pdf]

Table1 Performance Evaluation of the ResNet-152 Model in Synovitis Classification

Metrics	Normal Group	Case Group
Precision	0.86	0.71
Recall	0.10	0.99
F1-score	0.19	0.83
Accuracy	0.10	0.99
AP	0.67	0.87
AUC	0.77	0.77
